# Occult Spinal Dysraphism in the Presence of Rare Cutaneous Stigma in a Neonate: Importance of Ultrasound and Magnetic Resonance Imaging

**DOI:** 10.1155/2013/468376

**Published:** 2013-05-23

**Authors:** Claudio Rodrigues Pires, Jane Marília Matos de Medeiros, Edward Araujo Júnior, Adriano Czapkowski, Sebastião Marques Zanforlin Filho

**Affiliations:** ^1^Teaching Center of Computed Tomography, Magnetic Resonance Imaging and Ultrasound (CETRUS), São Paulo, SP, Brazil; ^2^Department of Obstetrics, Federal University of São Paulo (UNIFESP), Rua Carlos Weber, 956 Apartment 113 Visage, Vila Leopoldina, São Paulo, SP, Brazil

## Abstract

Occult spinal dysraphism is defined as a group of dystrophic conditions below an intact cover of dermis and epidermis. Ultrasonography using linear transducers is a fast, inexpensive, and effective method that makes it possible to view the content of the vertebral canal and bone structures. Magnetic resonance imaging (MRI) is reserved for elucidating the type of dysraphism and for planning corrective surgery. We present a case of a five-day-old female neonate who presented cutaneous stigmas (in the lumbar region, hands, and feet), in whom ultrasonography demonstrated dysraphism in the lumbar region. MRI confirmed the type of dysraphism and enabled surgical planning.

## 1. Introduction

Spinal dysraphism is a term used for a group of disorders characterized by incomplete fusion or lack of fusion of midline structures during the fourth week of embryogenesis [1]. The incidence of these defects shows significant geographical variation (from 0.5 to 5 per 1,000 births) and affects black individuals less frequently. Its prevalence is greater among females and in poor people [2].

Based on the physical findings, cases of spinal dysraphism can be grouped into two categories: open spina bifida, with protrusion of posterior neural tissue through a defect in the vertebra, which results in a lesion that is not covered by skin, such as meningomyelocele, and occult spinal dysraphism [3]. 

Occult spinal dysraphism is defined as a group of dysraphic conditions present below an intact cover of dermis and epidermis. It is therefore more difficult to diagnose on antenatal ultrasonography. It may be suspected in asymptomatic newborns because it is generally associated with abnormalities of the adjacent skin, such as cutaneous stigmas, hemangiomas, hair tufts, cutaneous appendices, sacrococcygeal dimples, and subcutaneous masses, particularly in the lumbosacral region [4]. The following abnormalities can be included as forms of occult spinal dysraphism: dorsal dermal sinus, spinal cord anchored by a lipoma, lipomyelomeningocele, diastematomyelia, and thickened filum terminale [5]. Lipomyelomeningocele is a form of spinal dysraphism in which the lipoma invades the dural sac, and it may involve the nerve roots and medullary cone [1]. In this abnormality, the spinal cord is low and anchored by the lipoma [2].

Ultrasonography has been used to evaluate the spinal canal since the 1980s [2]. The incomplete ossification of the posterior elements of the more caudal vertebrae in children of up to five or six months of age provides a good acoustic window for viewing the content of the vertebral canal and the bone structures [5]. Echography is considered to be an effective low-cost noninvasive method and plays a critical role in diagnosing or ruling out occult spinal dysraphism at birth [2].

Magnetic resonance imaging is another diagnostic imaging method for evaluating cases of occult spinal dysraphism. In T1 and T2 views, this technology enables detailed evaluation of the skin, medullary, canal and intervertebral discs, thus making adequate planning for corrective surgery possible [6].

Here, we present a case of a five-day-old neonate with occult dysraphism of lipomyelomeningocele type who presented cutaneous stigmas, and we demonstrate the main ultrasonographic and magnetic resonance findings from the spine. 

## 2. Case Report

**Figure 1:**
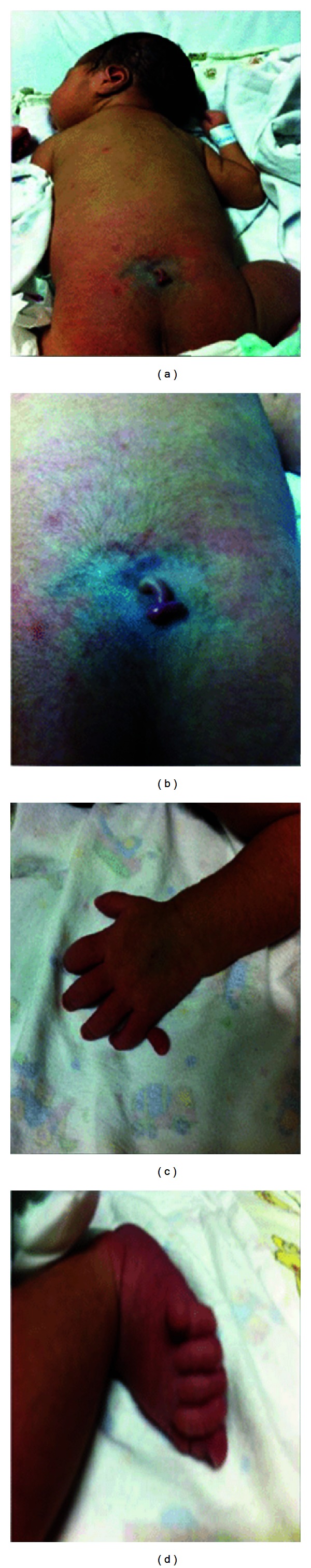
(a) and (b) Cutaneous stigma: skin appendage resembling a tail and violaceous maculae; (c) postaxial polydactyly of the hand; (d) postaxial polydactyly of the feet.

A five-day-old white female neonate was sent to our service for transfontanellar ultrasonography to be performed, because of the presence of cutaneous stigmas in the lumbar region. Physical examination on the newborn showed a skin appendage resembling a tail, on the midline in the lumbosacral region. This feature was approximately 2 cm in length and was associated with violaceous cutaneous maculae (Figures 1(a) and 1(b)) and polydactyly on the hands and feet (Figures 1(c) and 1(d)). A neurological examination done earlier had not shown any abnormalities. 

**Figure 2:**
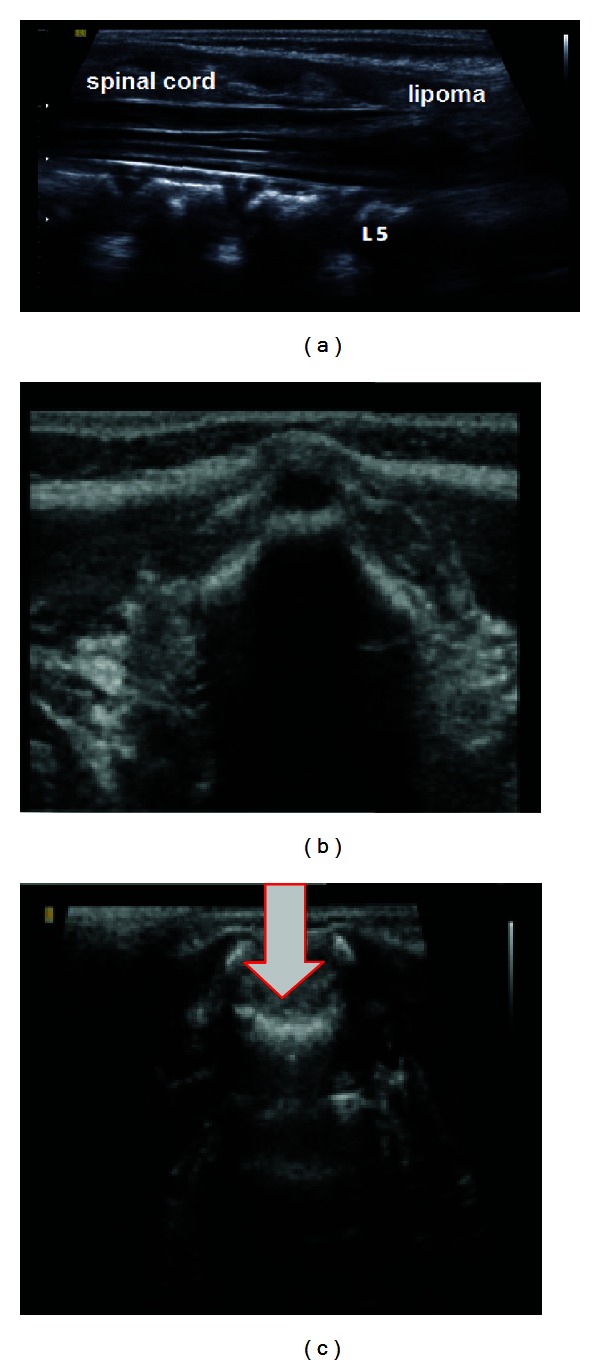
(a) Ultrasonography in the sagittal plane, showing a medullary cone extending as far as the sacral vertebrae, with a hypoechoic solid formation inside the canal (lipoma); (b) ultrasonography in the axial plane at the level of the upper lumbar spine (L1), showing a closed medullary canal; (c) ultrasonography in the axial plane at the level of the lower lumbar spine (L5), showing discontinuity of the posterior bone layers (white arrow).

The ultrasonographic examination was performed using the SonoAce X8 apparatus (Samsung-Medison, Seoul, Korea), with a multifrequency linear transducer (5–12 MHz). Images in B and color Doppler modes were obtained. The patient was firstly positioned on her mother's lap to perform transfontanellar ultrasonography and then in ventral decubitus to perform ultrasonography on the spine. Sweeps in longitudinal and transverse planes were performed, with the aims of making a detailed assessment of the contiguity of the anatomical features with the medullary canal: the outline and position of the spinal cord, the paraspinal musculature and the overlying skin. The ultrasonographic findings from the spine were (1) discontinuity of the posterior bone layers in the L5 and S1 projections, with an intracanal solid formation presenting undefined margins and a heterogenous hyperrefringent interior, adhering to the distal segment of the medullary cone; (2) a medullary cone extending beyond the L3 body (Figure 2). The diagnostic hypothesis was that a defect of the medullary canal was present in the region of the cutaneous stigma, with anchored spinal cord and an intracanal solid medullary formation with apparent peripheral invasion that was continuous with the spinal cord, suggestive of a lipoma. Transfontanellar ultrasonography did not show any abnormalities.

**Figure 3:**
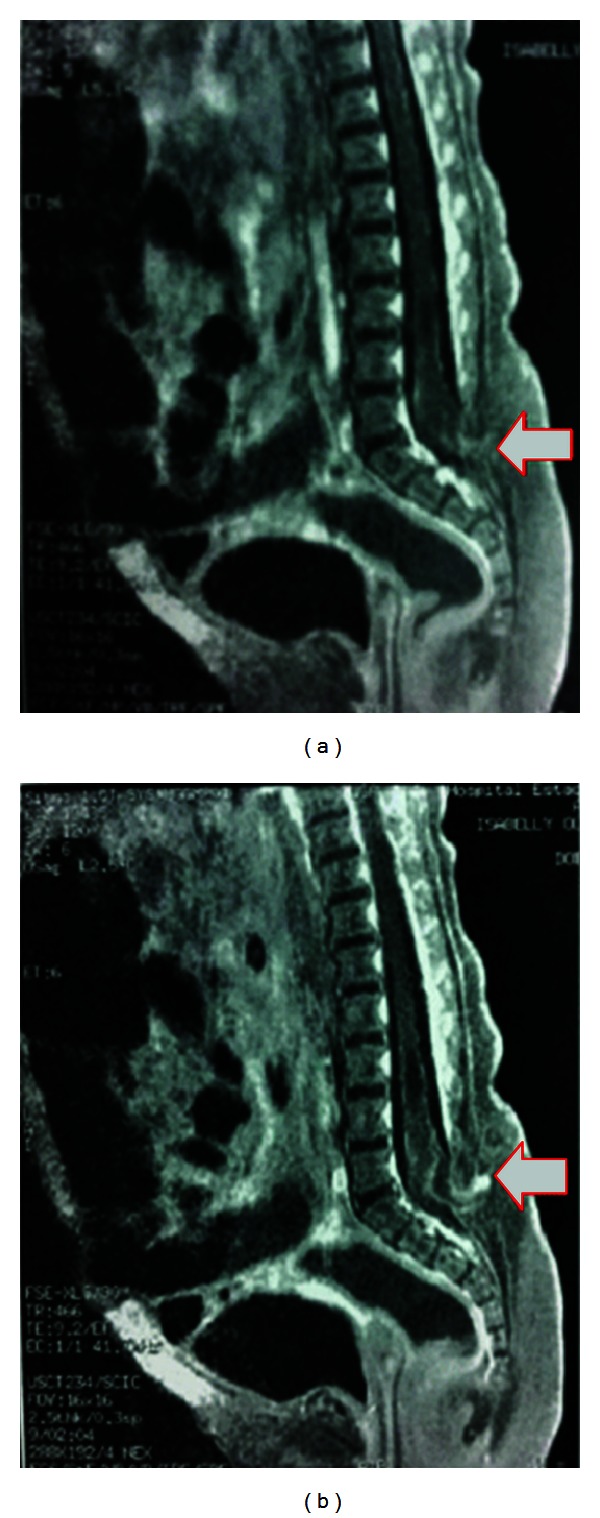
(a) and (b) Magnetic resonance imaging on the spine in the sagittal plane, showing a closure defect of the medullary canal at the level of the sacral region (S1) (white arrows), along with the presence of a posterior image with hypersignal in T1 and T2, compatible with a lipoma (red arrows).

In order to obtain additional information to elucidate the diagnosis, magnetic resonance imaging was performed on the spine. This produced the following findings: (1) a posterior fusion defect of the sacral spine, noting posterior herniation of length less than 1 cm, containing meninges, spinal fluid, and nerve structures at S1 level (myelomeningocele), which was in communication with the skin through a narrow connecting passage of length 1.5 cm (dermoid sinus); (2) signs of trapped spinal cord, with a low medullary cone down to S1 level, in association with a posterior image presenting hypersignal in T1 and T2, compatible with a lipoma, which also presented herniation with the myelomeningocele mentioned above (Figure 3); (3) central hydromyelia at the levels L3 and L4; (4) aligned vertebral bodies, with preserved morphology, outlines, heights, and signs; (5) intervertebral discs with heights and signs preserved, without evidence of hernias, protrusions, or bulges; (6) conjugation foramens with preserved amplitudes.

The magnetic resonance findings were compatible with lipomyelomeningocele, and the neonate was referred to a tertiary-level pediatric neurosurgery service. The neonate underwent corrective surgery and, over a six-month follow-up, presented normal neuropsychomotor development. 

## 3. Discussion

Cases of spinal dysraphism are rare, even in newborns with cutaneous stigmas. In three prospective studies in the literature, the highest incidence reported, in an evaluated population of 2,010 patients, was 7.2%, that is, 144 newborns who presented cutaneous stigmas. Of these, only 5.5% (8) were diagnosed with occult spinal dysraphism [2]. Some stigmas have been proven to present greater risk of occult spinal dysraphism, such as deep or atypical sacrococcygeal dimples, hemangiomas, cutaneous aplasia, subcutaneous masses, and exophytic skin lesions such as tails and hair tufts. Cases of multiple stigmas comprise another group at risk [7]. In our case, the neonate presented exophytic skin lesions in the lumbar region and hands (postaxial polydactyly). 

In our service, the protocol to neonates with high risk of occult spinal dysraphism with cutaneous stigma is accomplishment of spinal ultrasound using the linear transductor. The ultrasound has great capacity to assess the vertebral canal. The magnetic resonance imaging has the capacity to identify the type and the level of lesion. This case was different from our protocol because the neonate was referenced to realize the transfontanellar ultrasound, and only after the observation of cutaneous stigma, the protocol of occult spinal dysrapism was performed. 

Early diagnosis of spinal dysraphism is very important in order to minimize the sequelae that occur in patients who are not diagnosed before the growth spurt, who may suffer neural disorders due to medullary ischemia. Ultrasonography is a fast, safe, noninvasive, and low-cost method, and it also presents good correspondence with the findings from magnetic resonance imaging. The echographic findings suggestive of occult spinal dysraphism include a low position for the medullary cone, bulbous medullary cone, thick filum terminale, dorsal attachment of the spinal cord, and loss of cardiorespiratory pulsatory movement of the spinal cord [8]. Magnetic resonance imaging should be reserved for patients with positive or inconclusive results from ultrasonography, for confirmation of the diagnosis and surgical planning, as reported in the present case, in which the ultrasonographic findings were fully confirmed by the magnetic resonance imaging.

Early diagnosing of occult spinal dysraphism prevents progressive neurological dysfunction. However, detecting this condition in neonates is difficult since the neurological signs in these patients are not apparent. Because of the possibility of irreversible sequelae through delayed diagnosis, a screening method for patients at high risk of occult spinal dysraphism becomes necessary [7]. High-resolution ultrasonography is a fast and accurate method for screening for occult dysraphic lesions. On the other hand, the patients' thermal instability and need for sedation and the high cost contribute towards making it impracticable to use magnetic resonance imaging for screening purposes. Hence, this method is reserved for situations in which abnormal findings are seen, or when the normal maturation of the skeleton limits the possibility of viewing the medullary canal [5]. The protocol to neonates with high risk of occult spinal dysraphism has demonstrated good results; however, the physicians should be aware of neonates with cutaneous stigma because of high incidence of occult spinal dysraphism. 

In summary, we have presented a case of a neonate with occult spinal dysraphism associated with cutaneous stigmas. High-resolution ultrasonography using a linear transducer made it possible to identify and characterize the lesion, and magnetic resonance imaging confirmed the type of lesion (lipomyelomeningocele) and enabled adequate surgical planning. We believe that ultrasonography should be performed on patients who are at high risk of occult spinal dysraphism, such as those presenting cutaneous stigmas, congenital abnormalities, or neurological alterations, as a means of early diagnosis, thereby avoiding neuropsychomotor sequelae later on.
